# Generation of Antibody-Producing Hybridomas Following One Single Immunization with a Targeted DNA Vaccine

**DOI:** 10.1111/j.1365-3083.2011.02639.x

**Published:** 2012-04

**Authors:** I Øynebråten, T-O Løvås, K Thompson, B Bogen

**Affiliations:** Centre for Immune Regulation, Department of Immunology, University of Oslo and Oslo University HospitalRikshospitalet, Oslo, Norway

## Abstract

The standard protocol for generating antibody (Ab)-producing hybridomas is based on fusion of plasmacytoma cells with Ab-producing B cells harvested from immunized mice. To increase the yield of hybridomas, it is important to use immunization protocols that induce a high frequency of B cells producing specific Abs. Our laboratory has developed a vaccine format, denoted vaccibody that promotes the immune responses towards the delivered antigen. The vaccine format targets antigens in a bivalent form to surface receptors on antigen-presenting cells (APCs). Here, we used the fluorescent protein (FP) mCherry as antigen and targeted it to APCs by use of either the natural ligand CCL3/MIP-1α or single-chain variable fragment specific for major histocompatibility complex class II. The vaccine format was delivered to mouse muscle as DNA combined with electroporation. By this procedure, we developed two monoclonal Abs that can be utilized to detect the FC mCherry in various applications. The data suggest that the targeted DNA vaccine format can be utilized to enhance the number of Ab-producing hybridomas and thereby be a tool to improve the B cell hybridoma technology.

## Introduction

The need for monoclonal antibodies (mAbs) both for scientific purposes and for treatment of patients is expanding. Since 1975, when Köhler and Milstein [1, 2] developed B cell hybridoma technology, several approaches have been tested with the aim to improve the immunization procedure and optimize the immune response [3]. The basic methodology is based on multiple injection of protein antigen with or without adjuvant. Different adjuvants have been tested, and the protocols have been shortened by introducing single-step immunization and by using delivery systems securing sustained delivery (liposomes, polymers and virus particles) [4, 5]. The latest development is multiplex immunization that includes injection of several protein antigens in one mixture together with adjuvant followed by boost immunization [3, 6, 7]. By use of this procedure, several mAbs with specificity to distinct antigens can be generated in one animal.

In other protocols, DNA encoding the antigen has been delivered into the muscle, peritoneum, spleen or veins, but only intrasplenic injection, when administering only a single dose, induced Abs and supported the generation of viable mAb-producing hybridomas [8, 9]. Furthermore, codon optimization, fusion of the antigen to immune stimulatory sequences, as well as co-delivery of plasmids encoding adjuvant sequences have been performed to enhance the responses following genetic (DNA) immunization [10]. DNA injection has also been potentiated by using electroporation immediately after delivery [11, 12] and by prime-boost strategies (DNA injection followed by injection of protein antigen) [13].

Although DNA immunization holds great promise, low immunogenicity is a challenge. To enhance immune responses, we have generated a novel type of immunoglobulin (Ig)-based molecules (denoted vaccibodies) that are homodimers. These molecules have been bivalently targeted to antigen-presenting cells (APCs) by N-terminal expression of single-chain variable fragment (scFv) specific for mouse major histocompatibility complex (MHC) class II [14], CD40 [15], Toll-like receptor 2 [16] or natural ligands such as chemokines (CCL3/MIP-1α and CCL5/RANTES) [17]. In the C-terminal end, the APC-targeted vaccine molecules carry large bivalent antigens with intact B cell determinants [14, 17] for induction of potent T and B cell responses. Similar to complete Abs [18], vaccibodies [14, 17] may be delivered by injection of plasmid DNA into the dermis or skeletal muscle followed by electroporation. Transfected cells synthesize and secrete vaccibodies that can be found in serum and that target APC [14].

In this study, we introduced the fluorescent protein (FP) mCherry [19] in the antigenic unit of the vaccibody molecule and utilized the natural ligand MIP-1α or scFv specific for MHC class II to target APCs. First, we generated mAbs against the antigen mCherry by two DNA immunizations performed with electroporation followed by a boost immunization with mCherry protein. Next, we tried to simplify the protocol by immunizing just once by delivering DNA into the muscle before electroporation. We observed that mCherry in the vaccibody format elicited a higher number of Ab-producing hybridomas compared with delivery of mCherry alone. The mAbs generated in the study were tested in different applications, and we found that they are usable in ELISA, Western blotting, immunocytochemistry and flow cytometry. Based on our findings, we suggest that the protocols for generating mAb-producing hybridomas can be shortened and improved by use of the targeted DNA vaccine format. We further hypothesize that the DNA vaccine format can overcome the challenges posed by weak and difficult-to-purify antigens.

## Materials and methods

### Mice

Female BALB/*c* mice were purchased from Taconic (Ry, Denmark) and were 6–10 weeks of age when immunized with the DNA vaccine constructs. The study was approved by the Norwegian Animal Research Committee (Oslo, Norway).

### Construction of vaccines

Construction of the vaccine format containing hinge1, hinge4 and C_H_3 exons from human Ig γ3 in the dimerization unit and mouse (m)MIP-1α, mMIP-1αC11S, scFv^I-E^ or scFv^NIP^ in the targeting unit has been described previously [14, 17]. The mCherry gene was kindly provided by Prof. R. Y. Tsien (University of California, San Diego) [19] and inserted into the antigenic unit of the vaccine format by use of specific primers including the *Sfi*I restriction enzyme site in the 5′ and 3′ end of the gene. (Primers: *Sfi*I restriction enzyme sites are underlined and the start and stop codons are indicated in bold. The mCherry codons are depicted in capital letters. Forward 5′-ta ggcctcggtggcctg **atg** GTG AGC AAG GGC GAG GAG GAT AAC ATG-3′. Reverse 5′ ta ggccctgcaggcc**tca** CTT GTA CAG CTC GTC CAT GCC GCC-3′). The vaccine format was subcloned into the pLNOH_2_ vector [20], and the vaccine protein was expressed under the control of the CMV promoter.

### Immunization

Plasmids were purified by use of EndoFree plasmid kit (Qiagen, Hilden, Germany) and diluted to concentrations of 0.5 or 1.0 μg/μl in 0.9% NaCl. The mice were anesthetized by Hypnorm Dormicum, their legs were shaved, and conductive gel was applied on the skin before injection of 50 μl DNA solution into each quadriceps femoris. Immediately after injection, electroporation was performed by use of the Elgen electroporator device equipped with a caliper electrode (Elgen; Inovio Biomedical Co., Blue Bell, PA, USA). In some experiments, re-immunization was performed on day 47 by injecting 50 μl of 1 μg/μl DNA encoding mCherry into each quadriceps before electroporation, followed by a boost immunization with an intraperitoneal injection of 100 μg sterile native mCherry protein diluted in 0.9% NaCl.

### Generation of B cell hybridomas

Spleens and draining (lumbar and sacral) lymph nodes (LN) were harvested under sterile conditions, and cells were isolated by gentle teasing into sterile phosphate-buffered saline (PBS) before fusion with mouse plasmacytoma cells (OURI cells) using polyethylene glycol 1500 (Roche, Mannheim, Germany) as previously described [1, 21]. After fusion, the cells were pipetted into 96-well trays and cultured in RPMI 1640 containing 10% foetal bovine serum (Life Technologies, Carlsbad, CA, USA) supplemented with hypoxanthine-aminopterin-thymidine (Sigma-Aldrich, St. Louis, MO, USA). After 14 and 19 days, the cell culture media were analysed by ELISA to identify colonies producing mCherry-specific Abs. Limiting dilution was performed on the positive colonies.

### Purification of anti-mCherry Abs by affinity chromatography

The hybridomas producing anti-mCherry Abs were cultured in roller bottles for 7 days before the supernatants were harvested. Supernatants were subjected to a protein A Sepharose column (GE Healthcare, Uppsala, Sweden), and the column was washed extensively. Bound IgG was eluted with Glycin-HCl, pH 2.7, and collected in fractions of 5 ml. Aliquots showing high absorbance (280 nm) were pooled and dialysed in PBS containing 0.05% azide.

### Biotin labelling of anti-mCherry Abs

Purified Abs were dialysed in 0.1 m NaHCO_3_, and the concentration of the Abs were adjusted to 1 mg/ml. N-hydroxy succinimide biotin (Sigma-Aldrich, St. Louis, MO, USA) was dissolved to concentration 1.3 mg/ml in dimethyl sulfoxide (DMSO), and 90 μl was added dropwise while stirring per milliliter Ab solution. The mixture was incubated for 2 h at room temperature with weak stirring before dialysis in PBS containing 0.05% azide.

### Plasmids encoding fluorescent proteins

mOrange in pcDNA3.1, mPlum and tdTomato were kindly provided by Dr. R.Y. Tsien (University of California, San Diego). mCherry, mPlum and tdTomato were cloned into a pcDNA3-derived expression vector (named pLNOH_2_) [20] by use of specific primers and PCR (Primers: The *Bsm*I and *Bam*HI restriction enzyme sites are underlined, the stop and start codons indicated in bold, and the gene codons are depicted in capital letters. 5′mCherry; ggtgtgcattcc **atg** GTG AGC AAG GGC GAG GAG GAT AAC ATG. 3′mCherry; ggtgggatcc**tca** CTT GTA CAG CTC GTC CAT GCC GCC G. 5′mPlum; ggtgtgcattcc **atg** CGG GGT TCT CAT CAT CAT CAT CAT CAT ATG GTG AGC AAG GGC GAG GAG GTC ATC AAG G. 3′mPlum; ggtgggatcc**tca** GGC GCC GGT GGA GTG GCG GC. 5′tdTomato; ggtgtgcattcc **atg** CGG GGT TCT CAT CAT CAT CAT CAT CAT GG. 3′tdTomato; ggtgggatcc**tca** CTT GTA CAG CTC GTC CAT GCC GTA C.). pDsRed-monomer-Hyg-C1 was from Clontech. The FP enhanced green fluorescent protein (EGFP) and enhanced yellow fluorescent protein (EYFP) were fused to Rab proteins, and the plasmids were kindly provided by Prof. Peter van der Sluijs (Utrecht University Medical Center, the Netherlands) and Prof. Jim Goldenring (Vanderbilt University School of Medicine, TN), respectively.

### Transfection

HEK293 cells were cultivated in 6-well plates or CC2-treated Lab-Tek II chamber slides (Nunc, New York, NY, USA) and transfected by DNA encoding FPs by use of Lipofectamine 2000 (Invitrogen, Carlsbad, CA, USA) and the protocol delivered by the manufacturer.

### Preparation of cell lysates

Supernatants were harvested, and the cells were washed two times in 2 ml PBS before the cells were lysed by either CytoBuster Protein Extraction Reagent (Novagen, Madison, WI, USA) and the protocol delivered by the manufacturer or 1% Nonidet P-40 (NP-40 in 50 mm Tris-HCl and 150 mm NaCl). Protease inhibitors (100× cocktail from Sigma and EDTA) was added to the lysis buffers.

### ELISA

Microtitre plates were incubated overnight with 75 μl/well of recombinant mCherry (1 μg/ml) or capture Ab (anti-mCherry clone 1 or 2, 1 μg/ml). Samples (50 μl/well) were incubated overnight followed by incubation with 75 μl/well of biotinylated detection antibodies [clone 1 or 2 (1 μg/ml) or class or subclass specific antibodies] (incubation time approximately 1.5 h) and alkaline phosphatase-conjugated streptavidin (1:3000, approximately 1.5 h) (GE Healthcare). P-nitrophenyl phosphate in diethanolamine buffer was developed for 10–60 min, and the absorbance was measured at 405 nm with a Tecan Sunrise Microplate Reader (Tecan Austria Gesellschaft, Salzburg, Austria). The endpoint titre was set to absorbance values 2 times the values measured in relevant negative controls.

### Western blot

The samples were diluted in SDS-containing buffer and subjected to 4–20% Tris-Glycine polyacrylamide gel (Invitrogen). Alternatively, to examine monomeric vaccine proteins, the samples were treated with β-mercaptoethanol for 3 min at 95 °C before electrophoresis. SeeBlue Plus2 Pre-Stained Standard (Invitrogen) was used to indicate the molecular weights. The proteins were blotted onto a polyvinyl fluoride membrane (Biorad, Hercules, CA, USA) by 100 V for 1 h at 4 °C. Dry milk and casein (5% and 1%, respectively) in 0.1% Tween-20 was used to block the membrane before incubation overnight at 4 °C with biotinylated Abs followed by incubation with streptavidin-conjugated HRP (GE Healthcare). The protein bands were developed by a chemiluminescent peroxidase substrate, Lumigen (GE Healthcare), and images were acquired by Kodak image station 2000R (Eastman Kodak, New Haven, CT, USA).

### Chemotaxis assay

Six hundred microliter medium (RPMI 1640 with 1% bovine serum albumin) containing recombinant mMIP-1α (positive control, purchased from R&D Systems, Minneapolis, MN, USA) or the vaccine protein (supernatant harvested from transfected HEK293) was added to the bottom wells of Transwell plates (5 μm pore size; Sigma). Then, 100 μl of 2 million ESb-MP cells (kindly provided by Dr. J. Van Damme, University of Leuven, Belgium) were added to the upper wells before incubation at 37 °C for 2 h. Cells that migrated to the bottom wells were counted by flow cytometry.

### Binding of [scFv^I-E^-mCherry]_2_ to MHC class II

Approximately 500,000 mouse fibroblasts (L cells) stably transfected with Eβ^d^Eα^k^ (CA36.2.1) or D^d^ (CA25.8.1) were incubated for 1 h at 4 °C with supernatants harvested from vaccibody-transfected HEK293. The bound vaccine molecules were detected by biotinylated anti-human C_H_3 (clone HP6017) followed by streptavidin-PE before analysis by flow cytometry (FACScalibur instrument with cellquest software; BD Biosciences, San Jose, CA, USA). Non-targeted vaccibodies were used as negative controls.

### Immunostaining protocols

Transfected HEK293 cultivated in Lab-Tek II chamber slides were submerged in PBS and fixed by use of 4% paraformaldehyde (PFA; pH 7.4) for 10 min before washing 2 × 5 min in PBS and submerged in water or alternatively fixed by use of methanol for 10 min and then air-dried. For immunostaining, the fixed monolayers were incubated with biotinylated anti-mCherry Abs (5 μg/ml) for approximately 2 h, then with Alexa488- or Cy3-conjugated streptavidin combined with 4′,6-diamidino-2-phenylindole (DAPI) for approximately 1 h in room temperature. The immunostained cells were examined using a confocal laser scanning microscope (Leica TCS, Heidelberg, Germany) equipped with an Ar (488 nm) and a He/Ne (543 and 633 nm) laser. A Plan apochromat ×100/1.4 or ×40/1.00 Fluotar oil objective was used (Leica), and the images were acquired sequentially by means of proprietary Confocal software (Leica).

### Cloning of variable regions

Total RNA was isolated from hybridomas using RNAble®kit (Laboratoire Eurobio, Courtabæuf, France). cDNA was prepared by use of C_H_2-2 specific primers. Next, variable regions were amplified by PCR, Pfu Turbo DNA polymerase (Stratagene, La Jolla, CA, USA) and degenerate primers designed for amplification of unknown variable regions (S. Wälchli and GÅ. Løset *et al.*, submitted). The PCR products were purified and ligated into zero Blunt vector (Invitrogen). Plasmid DNA was purified, and the sequencing results were analysed using the current IMGT/V-quest (http://www.imgt.org/textes/vquest/) and NCI-Blast tools.

## Results

### Characterization of the dimeric vaccine molecule

**Figure 1:**
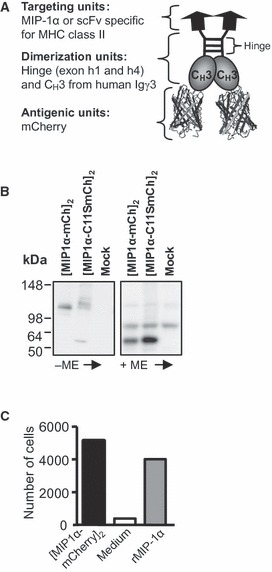
Characterization of the vaccine constructs containing mCherry. (A) Schematic drawing of the vaccibody protein, which is a homodimer consisting of three functional units: the targeting, the dimerization and the antigenic units. (B) SDS-PAGE (4–20% Tris-Glycine gel) and Western blotting of supernatant harvested from HEK293 transfected with vaccibody DNA. Negative control: supernatant from mock-transfected HEK293. The supernatants were reduced (+ME, mercaptoethanol) or not (-ME) prior to SDS-PAGE. Blotted vaccine proteins were detected by anti-mCherry and chemiluminescence. (C) mMIP-1α in the vaccine format is chemotactic. The indicated vaccibodies were added to the bottom wells of Transwell plates, and the number of CCR1^+^ CCR5^+^ Esb-MP cells migrating through the membrane within 2 h was determined by flow cytometry.

Construction of the homodimeric vaccine (vaccibody) has been described previously [14, 17]. The vaccine format consists of (1) a targeting unit containing either mouse (m)MIP-1α specific for the chemokine receptors CCR1, 3 and 5 or scFv^I-E^ specific for MHC class II (I-E^d^) (2) a dimerization unit composed of hinge + Ig C_H_3 (from human Ig γ3) and (3) an antigenic unit where we inserted mCherry (Fig. 1A). As negative, non-targeted controls, we utilized mutated mMIP-1α (mMIP-1αC11S) that is unable to bind the chemokine receptors or scFv^NIP^ that binds the hapten NIP (5-iodo-4-hydroxy-3-nitrophenacetyl). Because of the dimerization unit, two polypeptide chains may homodimerize into bivalent vaccine molecules.

To verify that the vaccine molecules were expressed and secreted, DNA was transiently transfected into HEK293 cells and the supernatants were harvested after 3–6 days and analysed. Western blotting revealed that the vaccine molecules could form dimers (Fig. 1B), and the observed sizes were similar to those estimated ([mMIP1α-mCherry]_2_ and [mMIP1αC11S-mCherry]_2_, about 110 kDa). Next, we wanted to verify that the targeting units were functional. By use of a chemotaxis assay employing the mMIP-1α-responding murine T cell lymphoma cell line Esb-MP, we observed that the cells migrated towards [mMIP1α-mCherry]_2_, verifying that mMIP-1α retained its chemotactic activity when inserted into the vaccine format (Fig. 1C). Moreover, Esb-MP cells migrated towards [mMIP1α-gp120]_2_ whereas only few cells migrated towards [mMIP1αC11S-gp120]_2_, confirming that the chemotactic activity was affected by mutation of Cys11 (I. Øynebråten A. B. Fredriksen, D. H. Barouch, and B. Bogen, manuscript in preparation).

**Figure 2:**
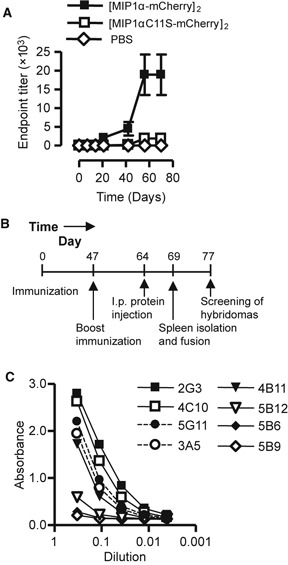
Ab responses, time schedule for immunization and screening of the fused hybridomas. (A) The vaccine constructs elicited mCherry-specific Abs. Targeted (mMIP-1α) or non-targeted (mMIP-1αC11S) vaccine constructs, or phosphate-buffered saline, were injected into the quadriceps of mice before electroporation. Serum samples were harvested at different time points after injection and analysed by an ELISA using mCherry as coat (*n* = 3 mice/group, error bars indicate SD). (B) BALB/*c* were given DNA encoding [MIP-1α-mCherry]_2_ i.m. immediately followed by electroporation. Mice with the highest level of anti-mCherry Abs were re-immunized (50 μg DNA in 50 μl NaCl per quadriceps), and given an i.p. protein boost before the spleens were harvested and fused with OURI cells to generate hybridomas. (C) Hybridomas were generated, and the cell culture supernatants were tested in different dilutions for their production of anti-mCherry Abs by an ELISA containing mCherry as coat.

Based on previous results [14, 17], we hypothesized that we could promote the Ab response towards mCherry by use of the targeted vaccine format. DNA encoding targeted (by mMIP1α) or non-targeted (mMIP1αC11S) was injected into quadriceps of BALB/*c* mice before electroporation. Serum samples were harvested, and the level of anti-mCherry IgG was determined by ELISA using mCherry as coat (Fig. 2A). Weak Ab responses were observed 3 weeks following immunization but were dramatically enhanced 8 weeks following immunization. Moreover, the targeted vaccine molecule elicited serum anti-mCherry IgG endpoint titres that were 8- to 9-fold higher than the endpoint titres induced by the non-targeted counterpart (Fig. 2A).

### Generation of anti-mCherry-producing hybridomas

Given the finding that the vaccine format could enhance Ab responses towards mCherry (Fig. 2A), we wanted to examine the possibility of generating mAb-producing hybridomas following delivery of DNA. DNA encoding [mMIP1α-mCherry]_2_ was injected into the quadriceps of BALB/*c* mice before the muscle was subjected to electroporation. The amount of anti-mCherry Abs in serum was determined at day 47 following immunization (Fig. 2B). The animal that induced the highest level of anti-mCherry Abs was immunized a second time with DNA encoding mCherry only. Finally, the mouse was given mCherry protein i.p. before the spleens were harvested and fused with a murine plasmacytoma (OURI cells) 5 days later (Fig. 2B). The hybridomas were screened for their ability to produce anti-mCherry mAbs by use of an ELISA with mCherry as coat. We identified eight hybridomas that produced anti-mCherry Abs, and five of these, in particular hybridomas 2G3, 4C10 and 5G11, generated large amounts of Abs (Fig. 2C). Following limiting dilution, two single-cell colonies were expanded and the mAbs denoted anti-mCherry clone 1 and clone 2 were purified by protein A affinity chromatography.

### Anti-mCherry clone 1 and 2 recognize several DsRed-derived FPs but not EGFP

**Figure 3:**
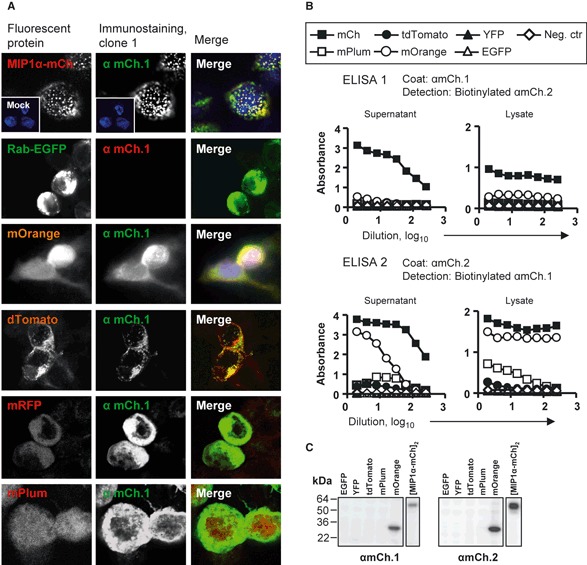
Specificity of the anti-mCherry Abs clone 1 and 2. (A) Microscopy revealed that the anti-mCherry Abs recognize the red-shifted fluorescent proteins (FPs) but not EGFP. HEK293 cells were transfected with DNA encoding various FPs, fixed and immunostained with biotinylated anti-mCherry clone 1 and visualized by Cy2- or Cy3-conjugated streptavidin. Fluorescence following transfection and immunostaining is shown in the left panel and middle panel, respectively. Merge images are shown in the right panel. (B) The specificity of the clones was examined by ELISA. Supernatants/cell media and cell lysates of transfected HEK293 were harvested and analysed in different dilutions (presented as log10 of dilution) by an ELISA. Clone 1 was used as coat and biotinylated clone 2 as detection antibody and vice versa. (C) The cell lysates analysed in B were also subjected to SDS-PAGE and Western blotting. The FPs were detected by anti-mCherry clone and chemiluminescence.

Fluorescent proteins may be fused to any target protein and provide a powerful tool to study the dynamics and the localization of proteins in live cells. To quantify and perform analysis on such fusion proteins, Abs towards the FP part may be useful in techniques such as ELISA and Western blotting. Therefore, we wanted to determine the specificity of the anti-mCherry mAbs. We chose to examine whether the mAbs could bind to EGFP, which has low sequence similarity with mCherry (Figure S1). We also included dsRed-derived FPs (which show high sequence similarity with mCherry, Figure S2): mOrange, dTomato, mRFP and mPlum, in addition to mCherry [19]. To perform these analyses, plasmids encoding the various FPs or [mMIP1α-mCherry]_2_ were transfected into HEK293 cells. Following incubation, the cells were fixed in PFA or methanol. Immunostaining with biotinylated anti-mCherry clone 1 or 2 followed by confocal analysis revealed that both mAbs recognized all the examined dsRed-derived FPs but not EGFP (Fig. 3A). Fixation in PFA induces intra- and intermolecular bridges and may reduce the antigenicity of proteins. However, similar staining intensities were obtained after methanol and PFA fixation, suggesting that the epitope is not immensely sensitive to fixation.

Next, to verify the aforementioned findings, we performed ELISA on lysates and supernatants from FP-transfected HEK293. The ELISA was set up in two different combinations; either clone 1 was used as coat together with biotinylated mAb clone 2 as detection (denoted ELISA-1) or *vice versa* (ELISA-2). ELISA-1 as well ELISA-2 resulted in high absorbance values for mCherry (Fig. 3B). Moreover, mOrange could be detected by both ELISAs. Somewhat different results were obtained for mPlum and tdTomato. They were detectable by ELISA-2, but not ELISA-1. EGFP were not detectable in any of the ELISAs, consistent with the observations following immunostaining (Fig. 3A,B).

Finally, the supernatants and lysates analysed by ELISA were subjected to SDS-PAGE and Western blotting. mCherry and mOrange were detectable when left untreated or treated by mercaptoethanol, whereas no band appeared for the other FPs (Fig. 3C and data not shown).

### Clone 1 and 2 contain different variable regions

Characterization of clone 1 and 2 revealed that they both were of subclass IgG2a. We next wanted to examine whether their variable regions differ and thereby could exhibit distinct affinities or recognize various FP epitopes. To address this issue, we isolated RNA from the hybridomas, the variable light and heavy chains were amplified by PCR and their sequences were determined. We found that clone 1 and 2 contain different heavy chain variable regions (Table 1). Consequently, the two mAbs may have different affinity and/or recognize distinct epitopes. However, this remains to be firmly established. Light chain of clone 2 could not be identified.

**Table 1:** Anti-mCherry clone 1 and 2 contain different variable regions. RNA was isolated from the hybridomas, and the variable regions were amplified and sequenced. Current IMGT/V-quest and NCI-Blast tools were used to identify the sequence.

	IGHV	IGHD	IGHJ
Heavy chain			
Clone 1	IGHV1-18*01 or 26*01 or 34*02	IGHD24*01	IGHJ3*01
Clone 2	IGHV 2-6-4*01	IGHD11*01	IGHJ4*01
			
	IGKV		IGKJ
			
Light chain			
Clone 1	IGKV1-110*01		IGKJ5*01

### Generation of anti-mCherry-producing hybridomas following a single DNA immunization

**Figure 4:**
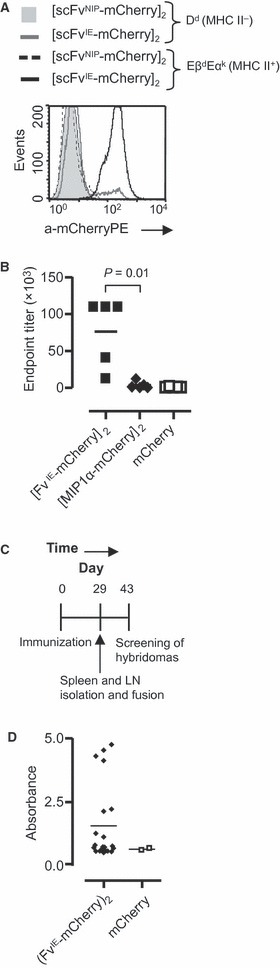
Targeting of mCherry towards major histocompatibility complex (MHC) class II enhanced the level of mCherry-specific Abs. (A) The targeting unit of [scFvI-E-mCherry]_2_ binds specifically to MHC class II. Supernatants harvested from transfected HEK293 were used to stain EβdEαk- or Dd-transfected L cells. The bound vaccine proteins were detected by biotinylated anti-mCherry, streptavidin-PE and flow cytometry. (B) BALB/*c* were given DNA encoding [scFvI-E-mCherry]_2_, [MIP1α-mCherry]_2_ or mCherry i.m. before electroporation. Blood was harvested after 14 days, and the titres of mCherry-specific IgG was determined by ELISA (*n* = 5 mice/group). (C) Time line. BALB/*c* were immunized as described previously, and draining lymph nodes (LN) and spleen were harvested for generation of hybridomas. (D) ELISA measurements of mCherry-specific Abs following fusion of draining LN with OURI cells. DNA encoding [scFvI-E-mCherry]_2_ or mCherry was injected into quadriceps of BALB/*c* before electroporation (*n* = 3 mice/group). After 29 days, the draining LN were harvested, fused with OURI cells and cultivated in 96-well plates. The cell supernatants were analysed in ELISA containing mCherry as coat.

Given the aforementioned results, we wanted to examine whether targeting of mCherry by use of the vaccibody format enhanced the number of Ab-producing hybridomas. Because data in our laboratory suggest that targeting of antigens to MHC class II is very efficient strategy when it comes to induction of Abs, we wanted to immunize BALB/*c* with DNA encoding [scFv^I-E^-mCherry]_2_. Western blotting of [scFv^I-E^-mCherry]_2_ from supernatants of transfected HEK293 revealed that it formed dimers and the size was similar to that estimated (approximately 145 kDa, Løvås *et al.*, in preparation). Moreover, [scFv^I-E^-mCherry]_2_ bound to mouse fibroblasts (L cells) transfected with CA36.2.1 (Eβ^d^Eα^k^) but not CA25.8.1 (D^d^), confirming that the targeting unit scFv^I-E^ bound specifically to MHC class II (Fig. 4A). DNA encoding the targeted vaccines or mCherry alone was delivered i.m. immediately followed by electroporation. Blood samples were harvested, and at early time points, targeting to MHC class II elicited higher Ab titres than targeting to chemokine receptors by use of MIP-1α and mCherry alone (Fig. 4B). Spleen and draining LN were harvested at day 29 after immunization, and the cells were fused with OURI cells (Fig. 4C). After 14 days, the cell supernatants were analysed by ELISA using mCherry as coat. No anti-mCherry-producing hybridomas were generated by use of the spleen (data not shown). However, when analysing the supernatant of the draining LN cells harvested from mice immunized with [scFv^I-E^-mCherry]_2_, we identified 21 wells of 95 wells that contained mCherry-specific Abs (Fig. 4D). By contrast, the draining LN cells from mCherry-immunized mice resulted in only two positive wells (of 95 wells, Fig. 4D). Examination of the cell culture also revealed that more hybridomas were generated following immunization with [scFv^I-E^-mCherry]_2_ compared to mCherry (data not shown). Finally, four and one anti-mCherry-producing hybridomas were developed from [scFv^I-E^-mCherry]_2_ and mCherry-immunized mice, respectively. Taken together, these data indicate that targeting of mCherry to MHC class II enhanced the number of activated B cells and the number of Ab-producing hybridomas. Moreover, early in the response, the draining LN rather than the spleen should be utilized for fusion and development of hybridomas.

## Discussion

A major aim in vaccine development is to elicit high levels of Abs in the immunized individuals. To accomplish this, several strategies have been tested and the most successful ones may be utilized to improve the protocols used for generation of mAbs. We and others have observed that targeting of vaccine antigens to surface molecules on APCs enhance T cell responses and the level of antigen-specific Abs in serum [14, 15, 17, 22–24]. In our laboratory, we have targeted several antigens to the chemokine receptors CCR1, 3 and 5 or to MHC class II by use of mMIP-1α or scFvI-E^d^, respectively. Both targeting strategies are successful in eliciting high levels of Abs (the present study and [14, 17]). In both cases, vaccines have been given as DNA combined with electroporation. We have here tested whether such targeted DNA immunization can enhance the number of hybridomas producing antigen-specific mAbs.

CCR1, 3 and 5 are expressed by APCs such as monocytes (CCR1, 5), dendritic cells (DCs) (CCR5), eosinophils and basophils (CCR3) [25], whereas MHC class II is predominantly expressed by professional APCs (i.e. B lymphocytes, cells of the monocyte–macrophage lineage and DCs) [26, 27]. The exact mechanism for why targeting of vaccine proteins to these surface molecules enhances Ab levels needs to be unravelled. However, we show that MIP-1α retains the chemotactic activity when expressed in the vaccine fusion format, and intramuscular delivery of DNA encoding MIP-1α is reported to result in infiltration primarily of DCs but also macrophages [28]. Targeting of antigens to MHC class II also results in infiltrates of APCs (T. O. Løvås *et al.*, in preparation). Thus, our targeting strategy appears to recruit APCs to the delivery site of the vaccine construct, enhance uptake and loading of APCs with the antigen and thereby promote stimulation of T cells that can help B cells. In addition, formation of APC-B cell synapses [29], where the targeted vaccine proteins form bridges between APC and B cells, could contribute. Finally, bivalency of the antigen in the homodimeric fusion proteins could promote cross-linking of B cell receptors and thus B cell activation.

Köhler and Milstein [1, 2] used the polyethylene glycol-induced fusion technology to generate mAbs. Later on, investigators have searched for alternative, simpler, time-saving strategies to increase the throughput of mAb production [10, 30]. However, the hybridoma technology is still the most widely employed method for making mAbs. To elicit antigen-specific Abs, animals are most often immunized with protein. In our first experiment, we immunized the mice twice with DNA and then performed a boost immunization with protein. This protocol resulted in several hybridomas secreting antigen-specific Abs, but the spleens were harvested approximately 2 months after the initial immunization. It would be preferable to shorten the immunization schedule prior to generation of hybridomas. In addition, a protocol depending on only one DNA immunization without the need of a protein boost, requiring protein production and purification, would be a step towards an easier and more convenient method. We observed that targeting to APCs induced high Ab levels already 2 weeks following immunization, and because the Ab levels lasted for several months, we hypothesized that one immunization would be sufficient to generate high numbers of B cells producing antigen-specific Abs. We further hypothesized that the traditional boost immunization 4 days prefusion could be omitted. This is so because DNA injection and electroporation will cause a sustained expression of the vaccine fusion protein for several weeks, and the continuous expression of the vaccine may keep the B cells activated. The results herein support such a simplified immunization preceding fusion. To immunize the mice, we used an electroporator from Inovio Biomedical. However, most likely, simple, ‘home-made’ electrodes would be sufficient when aiming to produce hybridomas.

Most protocols harvest the spleen and fuse splenocytes with the plasmacytoma cells. However, when harvesting the mice 4 weeks after one single DNA immunization, we found that fusion of draining LN cells with plasmacytoma cells generated Ab-producing hybridomas, whereas no hybridomas were induced when using the splenocytes. These findings may be explained by the time point we harvested the LNs. In addition, targeting of the vaccine protein may trap the antigen locally and in the draining LN and thus limit the systemic spreading of the antigen.

It is often time consuming and difficult to produce purified proteins for immunization. DNA immunization eliminates this problem as the vaccine is genetically generated and it is easy to produce sufficient amounts of plasmids for injection. DNA immunization also removes the worry of contaminants in the immunogen. However, a problem might be lack of secretion of vaccine proteins from the transfected cells. In our experience, secretion of vaccine fusion proteins from transfected cells is obtained in >90% of cases with a diverse collection of targeting units and antigenic units (B. Bogen *et al.*, unpublished oberservation).

Following delivery of DNA encoding [mMIP1α-mCherry]_2_ and a boost immunization with mCherry protein, two hybridomas producing large amounts of mCherry-specific mAbs were characterized. We found that the mAbs are useful in applications where the dsRed-derived FPs are present in a native conformation such as in ELISA and immunostainings. PFA induces covalent cross-linking and could potentially mask epitopes, but we found that the two mAbs recognized PFA- and methanol-fixated FPs to an equal extent. Following SDS-PAGE, only mCherry and mOrange were detectable. SDS acts as a dissociating agent, and polypeptides are assumed to become rod-like structures with a net negative charge per unit length. Thus, the mAbs may to some extent recognize conformational-dependent epitopes and/or be sensitive to charge. In all experiments, binding of the mAbs to various FPs were tested by use of cell lysates or supernatants harvested from transfected HEK293. It should be noted that the FPs might be expressed at different levels following transfection, meaning that we cannot draw a conclusion when it comes to the affinity of the mAbs for the different FPs. Based on fluorescence microscopy, tdTomato was expressed to a lower extent than mCherry and mOrange. EGFP and yellow fluorescent protein (YEP) were expressed at high levels and should be detectable by specific Abs.

Fluorescent proteins are folded into a β-barrel and consists of 11 β-strands that form a compact cylinder or barrel encircling a distorted α-helix containing the chromophore, and the chromophore is buried and hidden inside the barrel [31]. The dsRed-derived FPs, in particular mCherry, mOrange, mPlum and mRFP, have high amino acid sequence similarity outside the chromophore. Thus, it was not surprising that the anti-mCherry Abs also recognized dsRed-derived FPs. EGFP has N- and C-terminal ends similar to those of mCherry examined here. Because the mAbs failed to detect EGFP, they are most likely raised against the β-barrel.

The N- and C-terminal ends of most FPs are rather flexible regions; therefore, FPs are relatively indifferent to N- and C-terminal fusions [19]. In the present study, mCherry was inserted as an N-terminal fusion into the C-terminal end of the vaccine format. Consistent with this, our experience is that several different viral and tumour antigens can be inserted into the C-terminal end of the vaccine format, with maintenance of expression and secretion (for example, gp120 and hemagglutinin (data not shown), scFv^315^ and scFv^A20^ and fragment C of tetanus toxin [14, 16, 17]). Thus, based on our observations, we believe that the vaccine format can be suitable for expression and secretion of a number of different proteins, also those that are difficult to produce. Moreover, we suggest that the vaccine format represents a convenient tool and an improvement of the technology used for generation of mAbs as one single DNA immunization was sufficient.

## Supporting Information

**Figure S1** Sequence alignments of mCherry, EGFP, and YFP. The alignment was performed by ClustalW. The asterisks indicate identical residues; two vertical dots highly conserved residues; and single dots indicate lesser conserved residues.

**Figure S2** Sequence alignments of dsRed-derived FPs. The alignment was performed by ClustalW. The asterisks indicate identical residues; two vertical dots highly conserved residues; and single dots indicate lesser conserved residues.
