# Supplementary material for: Generation of Antibody-Producing Hybridomas Following One Single Immunization with a Targeted DNA Vaccine
Source: Scand J Immunol. 2012 Apr;75(4):379–88. doi: 10.1111/j.1365-3083.2011.02639.x (PMC3417379; doi:10.1111/j.1365-3083.2011.02639.x)
Supplement: Supplementary file 2 [file sji0075-0379-SD2.pdf]

Supplemmentary, Figure 1.

```
mCherry   MVSKGEEDNMAIIKEFMRFKVHMEGSVNGHEFEIEGEGEGRPYEGTQTAK 50
YFP       MVSKGEE----LFTGVVPILVELDGDVNGHKFSVSGEGEGDATYGKLTLK 46
EGFP      MVSKGEE----LFTGVVPILVELDGDVNGHKFSVSGEGEGDATYGKLTLK 46
          *******    ::. .: : *.::*.****:*.:.***** .  *. * *

mCherry   LKVTKGGPLPFAWDILSPQFMYGSKAYVKHPADIP--DYLKLSFPEGFKW 98
YFP       FICTTG-KLPVPWPTLVTTFGYGLQCFARYPDHMKQHDFFKSAMPEGYVQ 95
EGFP      FICTTG-KLPVPWPTLVTTLTYGVQCFSRYPDHMKQHDFFKSAMPEGYVQ 95
          :  *.*  **..*  * . : ** :.: ::* .:   *::* ::***:

mCherry   ERVMNFEDGGVVTVTQDSSLQDGEFIYKVKLRGTNFPSDGPVMQKKTMGW 148
YFP       ERTIFFKDDGNYKTRAEVKFEGDTLVNRIELKGIDFKEDGNILGHK-LEY 144
EGFP      ERTIFFKDDGNYKTRAEVKFEGDTLVNRIELKGIDFKEDGNILGHK-LEY 144
          **.: *:*.*  ..  : .::.. :: :::*:* :* .** :: :* : :

mCherry   EASSERMYPEDGALKGEIKQRLKLKD------GGHYDAEVKTTYKAKKPV 192
YFP       NYNSHNVYIMADKQKNGIKVNFKIRHNIEDGSVQLADHYQQNTPIGDGPV 194
EGFP      NYNSHNVYIMADKQKNGIKVNFKIRHNIEDGSVQLADHYQQNTPIGDGPV 194
          : .*..:*   .  *. ** .:*::.          *   :.*  .. **

mCherry   QLPGAYNVNIKLDITS-HNEDYTIVEQYERAEGRHSTGGMDELYK 236
YFP       LLPDNHYLSYQSALSKDPNEKRDHMVLLEFVTAAGITLGMDELYK 239
EGFP      LLPDNHYLSTQSALSKDPNEKRDHMVLLEFVTAAGITLGMDELYK 239
           **. : :. :  ::.  **.   :   * . .   * *******
```

Supplementary Figure 1. Sequence alignments of mCherry, EGFP, and YFP. The alignment was performed by ClustalW. The asterisks indicate identical residues; two vertical dots highly conserved residues; and single dots indicate lesser conserved residues.

Supplemmentary, Figure 2.

```
mCherry        MVSKGEEDNMAIIKEFMRFKVHMEGSVNGHEFEIEGEGEGRPYEGTQTAK 50
mOrange        MVSKGEENNMAIIKEFMRFKVRMEGSVNGHEFEIEGEGEGRPYEGFQTAK 50
mPlum          MVSKGEE----VIKEFMRFKVRMEGSVNGHEFEIEGEGEGRPYEGTQTAK 46
mRFP           MAS-SED----VIKEFMRFKVRMEGSVNGHEFEIEGEGEGRPYEGTQTAK 45
tdTomato       MVSKGEE----VIKEFMRFKVRMEGSMNGHEFEIEGEGEGRPYEGTQTAK 46
               *.* .*:    :*********:****:****************** ****

mCherry        LKVTKGGPLPFAWDILSPQFMYGSKAYVKHPADIPDYLKLSFPEGFKWER 100
mOrange        LKVTKGGPLPFAWDILSPQFTYGSKAYVKHPADIPDYFKLSFPEGFKWER 100
mPlum          LKVTKGGPLPFAWDILSPQCMYGSKGYVKHPADIPDYLKLSFPEGFKWER 96
mRFP           LKVTKGGPLPFAWDILSPQFQYGSKAYVKHPADIPDYLKLSFPEGFKWER 95
tdTomato       LKVTKGGPLPFAWDILSPQFMYGSKAYVKHPADIPDYKKLSFPEGFKWER 96
               *******************  ****.*********** ************

mCherry        VMNFEDGGVVTVTQDSSLQDGEFIYKVKLRGTNFPSDGPVMQKKTMGWEA 150
mOrange        VMNFEDGGVVTVTQDSSLQDGEFIYKVKLRGTNFPSDGPVMQKKTMGWEA 150
mPlum          VMNFEDGGVVTVTQDSSLQDGEFIYKVKLRGTNFPSDGPVMQKKTMGWEA 146
mRFP           VMNFEDGGVVTVTQDSSLQDGEFIYKVKLRGTNFPSDGPVMQKKTMGWEA 145
tdTomato       VMNFEDGGLVTVTQDSSLQDGTLIYKVKMRGTNFPPDGPVMQKKTMGWEA 146
               ********:************ :*****:******.**************

mCherry        SSERMYPEDGALKGEIKQRLKLKDGGHYDAEVKTTYKAKKPVQLPGAYNV 200
mOrange        SSERMYPEDGALKGEIKMRLKLKDGGHYTSEVKTTYKAKKPVQLPGAYIV 200
mPlum          SSERMYPEDGALKGEMKMRLKLKDGGHYDAEVKTTYMAKKPVQLPGAYKT 196
mRFP           STERMYPEDGALKGEIKMRLKLKDGGHYDAEVKTTYMAKKPVQLPGAYKT 195
tdTomato       STERLYPRDGVLKGEIHQALKLKDGGHYLVEFKTIYMAKKPVQLPGYYYV 196
               *:**:**.**.****::  *********  *.** * ********* * .

mCherry        NIKLDITSHNEDYTIVEQYERAEGRH---------STGG----------- 230
mOrange        GIKLDITSHNEDYTIVEQYERAEGRH---------STGG----------- 230
mPlum          DIKLDITSHNEDYTIVEQYERAEGRH---------STGA----------- 226
mRFP           DIKLDITSHNEDYTIVEQYERAEGRH---------STGA----------- 225
tdTomato       DTKLDITSHNEDYTIVEQYERSEGRHHLFLGHGTGSTGSGSSGTASSEDN 246
               . *******************:****         ***.

mCherry        ----MDELYK 236
mOrange        ----MDELYK 236
mPlum          -------LYK 229
mRFP           ----------
tdTomato       NMAV------ 250
```

Supplementary Figure 2. Sequence alignments of dsRed-derived FPs. The alignment was performed by ClustalW. The asterisks indicate identical residues; two vertical dots highly conserved residues; and single dots indicate lesser conserved residues.
